# Assessing the Emergency Response Role of Community-Based Organizations (CBOs) Serving People with Disabilities and Older Adults in Puerto Rico Post-Hurricane María and during the COVID-19 Pandemic

**DOI:** 10.3390/ijerph19042156

**Published:** 2022-02-14

**Authors:** Alina Engelman, Mariana T. Guzzardo, Marley Antolin Muñiz, Laura Arenas, Aracely Gomez

**Affiliations:** 1 Department of Public Health, California State University, East Bay, Hayward, CA 94542, USA; mantolinmuniz@horizon.csueastbay.edu (M.A.M.); lgomez20@horizon.csueastbay.edu (L.A.); agomez124@horizon.csueastbay.edu (A.G.); 2 Department of Human Development and Women’s Studies, California State University, East Bay, Hayward, CA 94542, USA; mariana.guzzardo@csueastbay.edu

**Keywords:** emergency response, community-based organizations, disability, older adults, Puerto Rico, COVID-19

## Abstract

In Puerto Rico, a host of factors makes the role of community-based organizations (CBOs) critically important in emergency preparedness and response (EPR) and disability-inclusive disaster risk reduction (DiDRR) addressing the needs of people with disabilities and older adults. The territory has been the site of recurring hurricanes, earthquakes, medical crises, and human-made disasters. Political, social, and economic problems unique to the archipelago have historically limited the preparedness and response capacity of governmental authorities, especially for its most at-risk populations. In a context of severe constraints on government resources, CBOs are positioned to play an outsized role in providing services for disabled and older adults before, during, and after emergencies. This study assesses the emergency preparedness and response capacity of CBOs (*n* = 22) for addressing the needs of people with disabilities and the elderly. Semi-structured, largely closed-ended interviews were conducted in Spanish with key informants at Puerto Rican CBOs. The interviews included questions about emergency preparedness and response training, as well as organizational capacity during COVID-19 and post-Hurricane María. This study posits that conditions in Puerto Rico place CBOs at the forefront of critical responsibilities including emergency preparedness and response, warranting assessment of their practices and resources to assist them in fulfilling their mission.

## 1. Introduction

In Puerto Rico, a host of factors makes the role of community-based organizations (CBOs) critically important in emergency preparedness and response (EPR) and disability-inclusive disaster risk reduction (DiDRR) addressing the needs of people with disabilities and older adults [1]. The territory has been the site of recurring hurricanes, earthquakes, medical crises, and other human-made and natural disasters. Political, social, and economic problems unique to the archipelago have historically limited the preparedness and response capacity of governmental authorities, especially for its most at-risk populations, including older adults and people with disabilities [2]. Puerto Rico suffers from its quasi-colonial status as a U.S. territory without representation in Congress, a debt crisis, exploitation of natural resources, the legacy of its use as a military testing ground, privatization of infrastructure, vulnerability to climate change, and widespread poverty [3]. The aim of this paper is to discuss how CBOs in Puerto Rico met the needs of these distinct populations in previous and current emergencies, as well as how this information about CBOs underscores their value in working toward more equitable outcomes in emergencies. Older adults and people with disabilities often have distinct needs in the context of emergency management, yet these needs can also overlap as older adults often also have a congenital or acquired disability.

In the context of severe constraints on resources, CBOs are positioned to play an outsized role providing essential services for disabled and older adults before, during, and after emergencies [4]. Substantial evidence exists that EPR efforts at the governmental level are not effectively reaching populations with functional and access needs [5]. Yet, research also demonstrated that during Hurricane María and earlier natural disasters, non-governmental organizations (NGOs), or CBOs, were often present and ready to provide support and assist with recovery long before the Federal Emergency Management Agency (FEMA) arrived. In response to Hurricane Katrina and Hurricane Rita in 2005, national and international NGOs and faith-based organizations were on the ground in the U.S. helping people evacuate, and providing furniture and housing, a week before FEMA arrived [6]. In the response to the COVID-19 crisis, there is evidence that CBOs filled a critical gap in the absence of a strong central government [7].

### 1.1. History and Effects of Hurricane María Prior to COVID-19

Hurricane María devastated the U.S. territory, leaving many without access to shelter, food, water, power, and adequate medical care. The effects of Hurricane María were compounded by disasters endured before and since, both natural and man-made, for example Hurricane Irma, a magnitude 6.4 earthquake occurring 3 months later, subsequent earthquakes, and the ongoing COVID-19 pandemic [8]. Furthermore, the effects of climate change will lead to continued natural disasters. The effects of these disasters are highlighted by Puerto Rico’s status as a U.S. territory, limiting the ability of the archipelago to receive support from the mainland U.S.

The original “disaster” inherent in the colonial relationship with the U.S., the privatization of health and social services, and a lack of infrastructure have also led to the pernicious effects of disaster capitalism in Puerto Rico [3]. The archipelago has often served the economic interests of the U.S. and investors, a situation further amplified in a post-disaster climate. For members of communities living with heightened medical and functional needs, such as individuals with disabilities, those with mental illness, and the elderly, the lack of investment in general infrastructure as well as emergency management and disaster preparedness have had detrimental impacts on livelihoods [9].

### 1.2. Positionality of Puerto Rico in the Aftermath of Disaster

As an unincorporated territory of the United States, Puerto Rico lacks self-determination and full representation in Congress, and residents of Puerto Rico are unable to participate in presidential elections [2]. In the context of disaster, colonial laws such as the Merchant Marine Act of 1920 and the Oversight, Management, and Economic Stability Act of 2016 in Puerto Rico limit the archipelago’s ability to respond appropriately to disasters, leaving the island with limited aid and attention compared to the mainland U.S. in terms of disaster response [2,10].

The Puerto Rico Oversight, Management, and Economic Stability Act (PROMESA), enacted in 2016 to address the economic crisis, imposes limitations on the spending ability of Puerto Rico’s government and public services. Furthermore, in the wake of Hurricane María, PROMESA significantly limited the local government of Puerto Rico by restricting the resources available to enable mobilization at a local level [2]. The board exerts control by increasing taxes while reducing public services and workers’ benefits, negatively impacting older adults with disabilities who frequently utilize these services.

In addition, Puerto Rico is extremely dependent on foreign production, with over 85% of Puerto Rico’s food supply being imported [11]. The impact of the COVID-19 crisis on global food production increased the risk of food insecurity across the archipelago. The Merchant Marine Act of 1920, also known as the Jones Act, limits the ability of non-U.S. vessels to engage in commercial trade with Puerto Rico, exacerbating the food insecurity already present by nearly doubling the prices of food throughout the archipelago, increasing Puerto Rico’s financial debt and limiting aid to only that available from the U.S. [2,12].

#### 1.2.1. Financial Crisis

Puerto Rico has always had a fluctuating, fragile economy. The same law that made Puerto Ricans U.S. citizens empowered the islands to raise money by issuing tax-exempt bonds, contributing to the growth of an unmanageable debt throughout Puerto Rico as some investors became tax-averse and eager to shelter income. As of 2018, Puerto Rico owed USD 100 billion in bonds and unpaid pension debts [13].

#### 1.2.2. Impact of Migration on the Prevalence of Older Adults with Disabilities

Due to a combination of economic crisis and disaster, there has been increased migration out of Puerto Rico to the mainland U.S., leaving the remaining population of Puerto Rico overwhelmingly older, facing increased rates of poverty and unemployment, and with lower levels of education [14]. In Puerto Rico, 18% of the population is aged 65 and over, while the national U.S. average is 15% [15]. The median household income throughout Puerto Rico is less than half of the U.S. national median household income, leaving 46% of Puerto Rico’s residents at income levels well below the federal poverty level [15].

As of 2016, nearly half of Puerto Rico’s elderly population reported at least one disability, with older adults of lower socioeconomic status at an increased risk of disability [16]. The prevalence of older adults with disabilities, combined with the widespread poverty and socioeconomic disadvantage throughout Puerto Rico, magnifies negative health outcomes in the wake of disaster.

#### 1.2.3. Puerto Rican Older Adults with Disabilities at Risk during Disaster

Research shows that the risk of adverse effects after emergencies increases among older adults with disabilities, particularly in the absence of governmental support. Older adults with physical or cognitive disabilities are disproportionately at an increased risk of injury, death, and developing post-disaster health problems [17]. In the aftermath of Hurricane Katrina, over 70% of the reported deaths were individuals over the age of 60, and in the aftermath of Hurricane Sandy nearly half of those who died were 65 or over [18].

Heightened mortality rates across Puerto Rico continued in the aftermath of Hurricane María [19]. This was largely due to preventable conditions stemming from a lack of quality infrastructure. Power outages led to the interruption of medical services and the inability to utilize electrically powered life-sustaining equipment. In addition, due to facility closures and damaged roads, many could not access health care or the transportation needed for evacuation [9]. After Puerto Rico was struck by Hurricane María, residents faced harrowing living conditions, including a lack of electricity, potable water, and appropriate housing that resulted in an extended humanitarian crisis [20]. Older adults and residents with disabilities were particularly underserved.

Some older adults with disabilities reported having no power until six to nine months following María. This was a life-threatening issue for individuals with complex medical needs such as those requiring ventilation machines, dialysis treatment, or refrigerated insulin. Furthermore, there continues to be a lack of appropriate medical services for people with serious physical and mental health conditions and disabilities, including services related to an emerging mental health crisis, which was underscored by a steady rise in suicide rates and suicide ideation following the hurricane [19,21].

#### 1.2.4. Factors Unique to Puerto Rico Impacting Emergency Response

In research examining the political determinants of disaster risk for people with disabilities in Puerto Rico, Morris et al. wrote, “First, there was the history of environmental degradation in Puerto Rico that exposed many in the population to health hazards [leading to chronic medical conditions] and that made people with disabilities at risk when disaster struck. Second, there was the failure to extend a modern safety net to the citizens of a U.S. territory that could protect against the most elementary forms of social risks, including those encountered by people with disabilities in a natural disaster” [20]. Therefore, it follows that access to public services for older adults with disabilities is likely to be more limited than it might be on the mainland U.S. Furthermore, Puerto Rico’s unincorporated territory status leaves older adults with disabilities with limited rights to control their own future and that of the archipelago.

#### 1.2.5. Rationale for Role of CBOs in Emergency Preparedness and Response

CBOs often focus on increasing access to health care, education, housing, food, and other essential resources through a wide variety of social programs and integrated information technology in the communities they serve. Thus, CBOs are key to the response and recovery efforts for underserved older adult and disabled populations. They are uniquely positioned to support preparedness and response efforts in the communities that they serve. Despite this scope, research has found that older adults may perceive the role of CBOs as being limited to, for example, obtaining information related to disaster evacuation, and they may seek more direct support from family and friends [22]. Therefore, it is critical that populations of older adults with disabilities recognize the wide range of EPR services they can benefit from and receive from CBOs.

#### 1.2.6. Purpose of the Article: Research Focus

The purpose of this research is to gain insight into the emergency preparedness role of CBOs and their challenges in the context of Hurricane María and COVID-19 as they pertain to the elderly, disabled, and mentally ill populations they serve. What is the emergency preparedness training and emergency response role of Puerto Rican CBOs serving older adults with disabilities? How can we use the findings to further social justice and to improve health and reduce health disparities in these two populations before, during, and after emergencies?

##### Rationale for the Article

- In Puerto Rico, the political climate, environmental injustices, and economic instability as a result of long-standing inequities have all contributed to the devastation and lack of governmental response in the aftermath of both Hurricane María and the COVID-19 pandemic. As a response to the humanitarian crisis throughout the archipelago, communities received support from CBOs to serve the needs of individuals with disabilities, older adults, and the mentally ill.
- The impact of emergencies in Puerto Rico provides evidence that colonialism is the major social determinant of health in Puerto Rico [2].
- Major disasters add new barriers to safety for individuals with disabilities that place them at greater risk than the general population [23]. As a result, it is imperative that people with disabilities have a structured plan in place, with the support of CBOs.

When assisting displaced evacuees, CBO staff are often faced with the difficult task of working with trauma-exposed clients while managing their own mental health needs. Research has identified a lack of adequate training and preparedness in CBO staff providing emergency services for the general population [24]. As can be imagined, lack of training can be a significant barrier to providing care in the form of physical and psychological assistance or supplying resources and basic needs, including housing, food, and medical care [25].

Furthermore, research has documented the fact that aid is often provided in the aggregate to entire populations rather than in the form of tailored, individual assistance that is necessary to effectively serve people with disabilities or other underserved populations [23].

This study posits that conditions in Puerto Rico invest CBOs with critical responsibilities regarding emergency preparedness and response for older adults with disabilities, warranting assessment of their practices and resources to assist them in fulfilling their mission.

## 2. Materials and Methods

Structured interviews were conducted in Spanish over the phone with key informants (KIs) at Puerto Rican CBOs. The interview questions addressed the role of CBOs during Hurricane María and the COVID-19 pandemic in supporting people with access and functional needs and older adults. It is key to note that the research team included members identifying as disabled, as Puerto Rican, and as part of the Puerto Rican diaspora. The interviews included questions about EPR training and organizational capacity. Institutional Review Board (IRB) approval was obtained from our university before collecting data.

### 2.1. Participants

The inclusion criteria for participating organizations concerned whether or not they served the elderly and/or people with a wide range of mental or physical disabilities. An extensive Internet search was performed to identify potential organizations meeting these criteria. After compiling a list of approximately 60 organizations, largely not-for-profit CBOs, a thorough recruitment effort was undertaken, including contacting the organizations and identifying KIs in a leadership position, to explain the project and request participation. Only three interviews took place prior to the COVID-19 pandemic, and the remaining 19 interviews took place during the COVID-19 pandemic. Therefore, most of the data collection occurred during the height of the COVID-19 pandemic.

While some of the organizations focused specifically on the needs of people with disabilities or older adults, other organizations served communities in need more broadly, including women, families, and young people in crisis or in particular local municipalities. This broad spectrum was included by design, in order to facilitate a deeper understanding of how major organizations can best serve older adults and people with disabilities in a time of crisis.

### 2.2. Data Collection Instrument

A structured interview was adapted from a survey used in a CDC-funded emergency preparedness project for the deaf, exploring the emergency preparedness capacity of CBOs as well as state- and territorial-level government officials [26]. The original survey was translated into Spanish and adapted for the purposes of the new research study in a Puerto Rican context. The structured interview consisted of primarily closed-ended questions, with six (6) open-ended questions. This gave the participating organizations the flexibility to share their perspectives on the challenges encountered and to make recommendations. This IRB-approved adapted version served as our data collection instrument and helped us collect both quantitative and qualitative data.

The questions explored the CBOs’ recovery efforts after the hurricane, as well as their training and experience in serving older adults and individuals with functional and access needs. It contained questions to assess the challenges faced before, during, and after emergencies, with special attention given to questions about the steps and measures taken to be better prepared for future emergencies. Open-ended questions provided insight into the challenges CBOs face during emergencies, their needs for support, and the major areas of concern regarding how the government has responded to emergencies in Puerto Rico. While the survey was intended to be about Hurricane María, KIs volunteered information about more recent and current emergencies, including earthquakes in Puerto Rico during 2020, as well as the COVID-19 pandemic.

Conducting the interview took approximately 30 to 60 min. Data were entered in real time by interviewers into the Qualtrics software, an online research tool that allows researchers to build and distribute surveys, as well as to analyze responses.

### 2.3. Sampling Frame

A sample of 22 senior- or disabled-serving CBOs was selected based on expert opinion from our research team, which includes researchers identifying as Puerto Rican as well as disabled researchers. These 22 senior- or disabled-serving CBOs had on average 7030 clients (median: 1145; range: 16–22,000 clients). Of the 22 organizations surveyed, 18 of them were local non-profit organizations in Puerto Rico, 1 was a non-profit organization in the U.S. and 3 were agencies/departments of the local government in Puerto Rico. Some CBOs only worked with older adults (e.g., those aged 65+) who primarily have age-related functional impairment. Other organizations work with a segment of the population with a specific disability, regardless of age. The sample included: (1) CBOs working specifically to address the needs of a particular disability or older adults and (2) CBOs serving a broader spectrum of Puerto Ricans facing a variety of crises including poverty, homelessness, food insecurity, and mental illness, as well as women or families-at-risk and homebound disabled older adults.

### 2.4. Data Analysis

For the closed-ended items, we analyzed the descriptive statistics using Qualtrics, which facilitated a summary of the responses in Excel. A team of three of the five authors performed the coding of the responses to the open-ended questions. They worked individually and then came together to discuss the codes. Our qualitative analysis entailed inductive coding similar to the steps of thematic analysis, with a focus on description and semantic meanings in the data [27]. We first familiarized ourselves with the data by reading through the narrative responses to the open-ended questions. Then, for each question, we coded the responses, with a focus on patterns. The codes were grouped to identify common themes across respondents. The decisions on common themes in the data were made as a team through group discussions.

## 3. Results

Open-ended responses highlighted the following themes: a need for mental health training for the elderly, mental health training for staff, more efficiency in emergency management, and the necessity of including people with disabilities in the official emergency plan. Respondents characterized the government as inefficient and lacking transparency, and also emphasized that their emergency plan lacked information on organizations that help specific populations, which reduced the effectiveness of the emergency response. Many of the KIs interviewed stated they would like to encourage the government to tap into the wealth of knowledge and trusted connections that CBOs and community leaders have formed in specific communities of people with disabilities and older adults. They also mentioned the need for the Attorney General to require COVID-19 vaccinations for the elderly. Additionally, many KIs mentioned the need for resilience and independence; however, solar, water, and overall infrastructure improvements must be made to enable this independence. Overall, KIs explained that more training was needed regarding various subgroups with functional and access needs.

### 3.1. Organizational Profiles

The CBOs sampled serve clients numbering in the hundreds to the tens of thousands per year. They provide a comprehensive range of services to particular subgroups of at-risk disabled and elderly populations such as the homeless, those with low incomes, those affected by food insecurity, the homebound, women and families in crisis, and those with mental health conditions.

#### 3.1.1. Demographics

CBOs (*n* = 22) estimated the number of clients serviced per year, which ranged from 50 per year to 22,000. Eight small CBOs served fewer than 500 clients a year, while four medium-sized CBOs served 500–1000 clients, seven large CBOs served 1000–4000 clients, and two very large CBOs served 4000–73,000 clients. One organization served 300 clients per day and had provided 40,000 meals since 2017.

**Figure 1:**
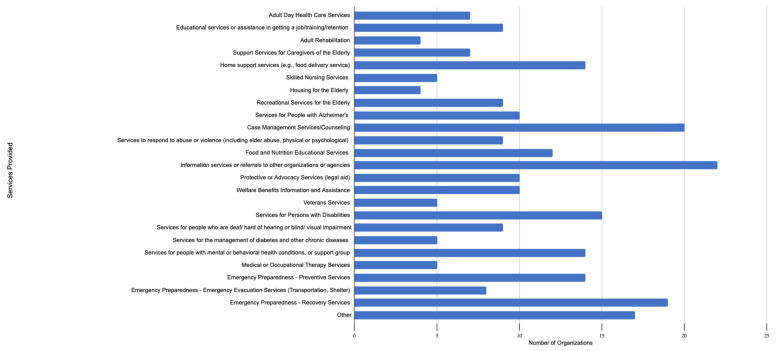
Comprehensive services provided to clients by Puerto Rican CBOs after Hurricane Maria and COVID-19. Figure 1 shows a comprehensive list of services typically provided, based on CBOs’ respective missions, as well as additional services to address community needs after Hurricane María and COVID-19.

A greater percentage of CBOs reported providing emergency response services compared to emergency preparedness services. Yet, the majority of CBOs provided a wide range of emergency services, including recovery services (86%, 19/22); evacuation services, including transportation and shelter (36%, 8/22); preventative services (64%, 14/22); and domestic violence response, including elder abuse; and physical or psychological abuse (41%, 9/22). In addition, CBOs provided critical basic needs including home support services and food delivery (64%, 14/22), food and nutrition education (55%, 12/22), welfare benefits assistance (45%, 10/22), and protective and advocacy services (45%, 10/22), along with housing, case management, and rehabilitation (Figure 1). The majority of CBOs undertook these response efforts even though such efforts were not part of their main mission.

#### 3.1.2. Populations Served

Sixty-eight percent of CBOs surveyed reported providing services for people with disabilities (15/22). This included offering medical or occupational therapy (18%, 4/22), services or support groups for people with mental or behavioral health conditions (64%, 14/22), and services for people who are deaf/hard of hearing or blind/visually impaired (41%, 9/22), as well as case management services and counseling (91%, 20/22).

The results showed that 95% (21/22) of organizations interviewed provided services to older adults aged 65+, and 82% (18/22) provided services to older adults aged 50–64. This included adult rehabilitation (18%, 4/22), services for people with Alzheimer’s disease (45%, 10/22), support services for caregivers of the elderly (32%, 7/22), skilled nursing services (23%, 5/22), housing for the elderly (18%, 4/22), and recreational services for the elderly (45%, 10/22).

The missions of the 22 organizations included the following key terms: the elderly (6), women (2), prevention of violence against women and youth, and families at risk or in crisis (7), people with disabilities including autism, intellectual disabilities, and the deaf and hard of hearing (6), and specific geographical areas including farming areas (3). Two organizations had the terms emergency or crisis response in their mission. One focused on emergency response and community restoration post-disaster. The other organization focused on families in crisis, with a mission to serve those with limited resources such as individuals facing natural disasters, economic constraints due to illness or disability, old-age, divorce, or loss of employment.

**Figure 2:**
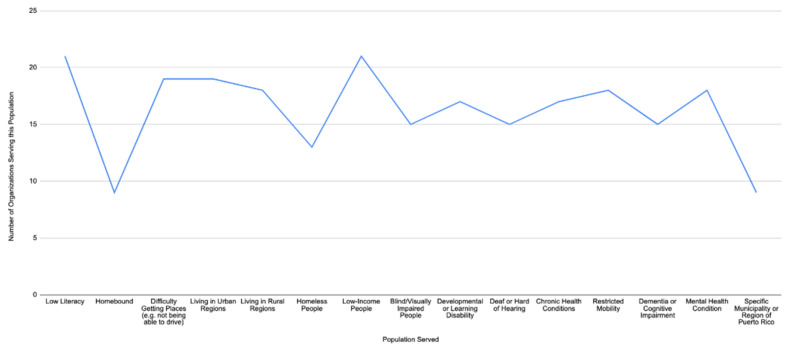
Populations served by Puerto Rican organizations (*n* = 22).

Most notably, despite the key populations mentioned in the missions of these CBOs (*n* = 22), these organizations often also served subgroups or other at-risk populations during an emergency. These included those identifying as homeless, low literacy (21/22 or 95%), home-bound, those with low income (21/22 or 95%), or those with chronic health conditions, reduced mobility, dementia, and mental health conditions (Figure 2 Populations Served by Puerto Rican Organizations).

### 3.2. Emergency Preparedness Training Provided

#### 3.2.1. Emergency Preparedness Training for Staff

In response to Hurricane María, the majority of CBOs (75%, 15/21) reported incorporation of emergency preparedness training for staff (excluding one organization that was established after Hurricane María). However, 29% (6/21) of organizations reported that no training was provided, and one CBO left this question unanswered.

#### 3.2.2. Frequency and Source of Training

Of the organizations providing emergency training for staff after Hurricane María (*n* = 15), two CBOs reported training once a year, three reported training several times a year, and three reported training just once after the hurricane. Most notably, almost half of the interviewees (7/15, 46.6%) did not respond to this question. Those who did reported receiving training ranging from a week prior to the interview to three years prior to the interview.

Some organizations facilitated, or were in the process of organizing, training in-house (*n* = 6), including two organizations providing mental health support to staff through their own counselors or psychologists. Others partnered with external organizations such as local federal agencies, non-profit organizations, and the University of Puerto Rico School of Medicine (*n* = 2) or attended emergency response conferences. Local and federal government agency partnerships included FEMA, the Municipality of San Juan, and the State Emergency Management. Non-profit organizations included minority- and disability-serving organizations including Peace for Women, TechSoup, UBAD Puerto Rico, Movimiento para el Alcance de Vida Independiente (MAVI), Centro Pro Vida Independiente (CEPVI), Sordos Unidos, SURT, Organizacion de Santa María, and Pueblos Unidos.

Interviewees reported that some of these emergency preparedness training sessions for staff provided specific information on supporting the elderly (*n* = 11, 50%), the deaf and hard of hearing (*n* = 8, 36.3%), the blind or visually impaired (*n* = 7, 31.8%), people with disabilities (*n* = 8, 36.3%), people with cognitive or developmental disabilities (*n* = 9, 40.9%), and people with mental health conditions (*n* = 11, 50%).

#### 3.2.3. Emergency Preparedness Training for Clients or Caregivers

It is notable that 50% (11/22) of the CBOs reported providing classes or training for clients or caregivers on emergency preparedness, while 50% did not (11/22).

### 3.3. Emergency Preparedness and Response Activities

For those organizations that provided services after Hurricane María, 16/18 (89%) of the CBOs mentioned providing information/referrals to other services, and 13/18 (72%) mentioned that they provided emergency or disaster updates.

#### Emergency Response Activities

A number of CBOs used volunteers to deliver hot food to elderly people without electricity in their homes (3). One very large CBO, serving a local municipality focused on community-building, stated that due to a community-wide loss of electricity and housing in the aftermath of Hurricane María, and thanks to volunteer support and donations, they distributed “water, food, repellents, hot food because many people could not cook, lacked insulin to inject, pampers, and games for children because there were no schools. The children were traumatized without water and electricity”.

Three CBOs provided supply distribution and donations as an emergency response effort, including clothing. Three CBOs reported offering lectures and education to their communities. Two disability-serving CBOs focused primarily on disability in their response efforts: one CBO stated that information was provided on how to assist individuals with disabilities during an emergency by MAVI, and one CBO offered interpretation for the deaf population in collaboration with FEMA. One CBO offered psychological services (trauma and crisis management) in their response efforts.

CBOs reported providing recovery services including financial assistance to clients facilitated through FEMA and PREMA (2), workshops (1), psychological help including trauma and crisis management and nutrition education (1), and donations from the Red Cross and Walmart to care for individuals with disabilities (1), and one CBO reported obtaining emergency response certification for its own organization (1).

### 3.4. Barriers after Hurricane María

**Figure 3:**
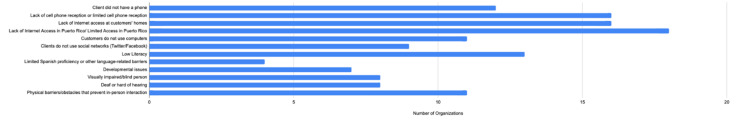
Barriers after Hurricane María (*n* = 22).

Findings show that in Puerto Rico’s post-disaster climate: (1) due to a lack of infrastructure there were serious communication and transportation problems, impeding delivery of supplies as well as the safety and health of clients and staff, (2) there was a need for more comprehensive and frequent EPR training in order to be better equipped to respond, (3) there was a lack of government support for CBOs, (4) a wide range of essential services were provided, sometimes going beyond the original scope of the organization even while operating under severe financial, logistical and resource constraints, and (5) there was a need for mental health and other support for staff and clients of CBOs (Figure 3).

CBOs reported that communication barriers and lack of Internet access created additional challenges in reaching populations already facing communication barriers such as low literacy, deafness and hardness of hearing, people not connected to social media, and people with limited Spanish reading or language proficiency due to a communication or developmental disability. In addition, homebound older adults faced major physical barriers in obtaining CBO assistance.

There were also communication and transportation barriers related to limited cell phone service and roads blocked by debris and fallen structures, which impeded the delivery of supplies. CBOs therefore had difficulty reaching their clients after the hurricane.

The lack of government support was highlighted by KIs reporting that the government did not provide emergency management information to them and were unwilling to work with them to provide services within communities. Overall, KIs reported that government officials were unresponsive to their requests for help or information. Regarding the fourth barrier, according to KIs, CBOs were working under financial, logistic, and resource limitations, partly because they were working far beyond the scope of their original mission. However, many KIs mentioned that financial support was an issue even before the hurricane.

Regarding the fifth barrier, KIs reported that their clients were facing major mental health issues, but they also emphasized that staff also needed mental health support. They noted that they were going through many of the challenges their own clients were facing after Hurricane Maria, including a lack of electricity and no or limited potable water, and therefore they also needed support.

#### Adjusting Services Due to Hurricane María

Findings from CBOs located in several regions of the main island demonstrated that they faced immense challenges, including a loss of electricity and water after Hurricane María. A small retirement home emphasized how their institution had suffered minimal damage after María, but the loss of electricity due to poor infrastructure had impacted them the most because they had bed-ridden patients who relied on life-supporting machines that functioned with electricity. One KI mentioned, “it affected us greatly because we have patients in beds, patients who need respirators, suction machines, a series of machines that run on electricity. Not having that service, nor the generator running, was very difficult". This dependence on electricity highlights the need to find alternatives such as encouraging more solar energy or restoring the state of the grid in Puerto Rico.

Organizations provided a wide range of services to clients after Hurricane María, most notably information and referrals, outreach in the home or neighborhood, food, emergency updates, and durable medical equipment including wheelchairs, crutches, and blood-pressure monitors, which are especially critical for people with disabilities and older adults (Table 1).

Multiple CBOs described making emergency preparedness improvements post-Hurricane María. One medium-sized organization serving families at risk mentioned that after Hurricane María, it began to prepare for disasters. This entailed looking for sponsorship to create a recovery resilience center and help support communities.

CBOs encountered challenges including a lack of communication, the necessity of undertaking door-to-door personal outreach, and the lack of resources and supplies. In addition, organizations had to adjust their goals after the disaster:

Our organization is not a specialized disaster management organization. After Hurricane María we have started to prepare for disasters and we are opening a resiliency center. Because of Hurricane María we have gotten sponsorships, and we have set up solar power to help the community. We have gotten funding to install a water well to service the community, and a radio communications system to support the community. After the disaster, what we did was to look for sponsorships to have a resilient recovery center...in a disaster and to support the community. (Medium-sized non-profit organization serving youth and families to prevent risk).

Organizations expanded the services they provided to communities after an emergency. For example, one stated “We do deliver food but not always. We do it in case of an emergency. When Hurricane María happened, we took care of bringing hot food to the people because there was no electricity, so they couldn’t make their own food”. (Very large non-profit organization serving a local municipality). In addition, they discussed how they stepped in when the government was not around: “We try, as everyone should, to always look for the physical addresses of those who need it the most and partner with them to provide services that the government doesn’t provide and that they can’t get...” (A small organization serving people with autism).

In response to a question about how organizations plan to check in on clients after future emergencies (Table 2), a very large non-profit organization serving a local municipality stated, “We work in alliance with organizations, we make a work committee and when something happens, we go to the street to help people. We went street-by-street and house-by-house because we had no electricity or resources. There was no electrical system or [any]where to connect”.

### 3.5. Adjusting Services Due to COVID-19

Although various barriers were encountered during Hurricane María, CBOs sought to rectify these issues during the COVID-19 pandemic. CBOs have restructured their services to minimize their exposure to COVID-19 while continuing to provide the support and services their clients need, even in the midst of severe resource constraints. Inspired by their experiences since Hurricane María and COVID-19, organizations reported planning on utilizing an even wider range of strategies to care for and monitor clients in future emergencies. The vast majority of CBOs intend to provide food and connect with clients through phone and social media in case of future emergencies (Table 2). Less commonly, organizations intend to provide first aid and CPR, provide shelter, provide sign language interpretation, provide transportation, and establish a buddy system.

It was found that organizations, including a large disability-serving non-profit organization, distributed emergency kits for the prevention of COVID-19, with face masks, vitamins, pamphlets, and hand sanitizer, and also provided food delivery to client homes. A very large non-profit organization serving a local municipality mentioned, however, the necessity of reducing the frequency of lunch distribution to minimize COVID-19 exposure. From 2017 to 2021 that organization had distributed 40,000 lunches to its surrounding communities.

CBOs’ wide range of existing, comprehensive, and direct services gave them a special ability to support their clients during COVID-19 while also reducing community transmission of COVID-19: “We have 52 elderly people who we give home delivery of food on weekends, housekeeping services, and nursing services". (Large community organization serving those on low incomes and the elderly). This large organization provides services to the community in general, particularly low-income elderly individuals and those facing homelessness, lack of education, unemployment, and drug abuse. They also provide services to unemployed women so that they can obtain employment.

During the COVID-19 pandemic, in addition to providing emergency kits for the prevention of COVID-19, organizations have also provided transportation and job training. A small retirement home stated that during the pandemic its staff had been able to provide services despite challenges:

We have not had any critical situation and the earthquakes, thank God, have not affected us physically because economically everything has affected us because our income has decreased drastically. At this moment, because we have a high-risk population, we don’t have many volunteers. No outsiders are allowed in or out. We only allow the employees of the home to come in, and that has helped us so far.

This quote underscores the resourcefulness of CBOs, as well as the funding and support needs that many others articulated.

### 3.6. Policy and Programmatic Recommendations

The majority of KIs reported that multiple actions were necessary to improve services to clients after a future emergency, including increasing the availability of supplies and equipment. More importantly, CBOs reported a need to improve the emergency preparedness training of the organization’s staff/employees (16/20, 80%), improve the emergency preparedness training of the organization’s clients (16/20, 80%), and improve educational materials in alternative formats for the deaf and hard of hearing and the blind (16/20, 80%). A significant percentage also recommended increasing the number of staff devoted to emergency preparedness (15/20, 75%) (Table 3). Further, government support was seen as essential: “In Puerto Rico, if the state becomes a facilitator and gives resources to community organizations, the aid would reach more people and would be more efficient”. (Medium-sized non-profit organization serving youth and families to prevent risk).

A number of recommendations were made for improving Puerto Rico’s emergency operations plan. CBOs implied that special populations of older adults with disabilities must be included in emergency planning:

Most of these emergency plans never contemplate this population (deaf community) and that they are treated differently because they have greater needs and are always a little behind. The same with the media; there is very little information and sign language for deaf communities. (Large non-profit organization serving some people with disabilities).

Therefore, there is a need to better address the needs of the deaf community. Additionally, a KI from a large non-profit organization serving at-risk youth explained the need “[t]o have a synchronized plan between the governmental institutions. By not having set all these communications prior, it made getting supplies/electricity/help to the people in Puerto Rico much more complicated”. (Large non-profit organization serving at-risk youth).

This KI emphasized the need for better collaboration between CBOs and local or federal government agencies addressing emergency management. Additionally, several KIs, including a small retirement home, mentioned the need to improve the infrastructure in order to “make all things better”:

They should talk to community leaders who are the ones who know where the faults are and what they need. There are thousands of community leaders who could make a specific emergency plan for the population because hurricanes can impact people in different ways. (Very large non-profit organization serving a local municipality).

CBOs expressed a need for greater investment in equipment and additional resources:

Increase the creation of community centers to become support centers, disaster recovery centers, that have their own electric power, solar, water, communications system and thus empower communities to respond to disasters and not depend on the government. (A medium-sized organization serving youth and families at risk).

#### 3.6.1. Mental Health Support for Staff and Clients

Organizations stressed the need for more comprehensive mental health support for staff as well as clients, given the particular challenges and stresses involved in post-emergency response and recovery: “In relation to mental health and in situations of emergencies, it is important to be effective and proactive in communication with the participants as their mental health is likely to deteriorate by the cause of such an emergency”. (Large non-profit organization serving at-risk youth). CBOs were frank about the challenges of providing mental health services after a disaster.

It was a bit difficult because of all the damage, there were 75 houses completely destroyed, 1200 roofs destroyed. There were people who could not physically leave their homes. We helped an elderly man who couldn’t get out. People needed more psychological help after the hurricane, so we referred people to where they could get that psychological help. Although it was difficult, we were able to help many people. (Very large non-profit organization serving a local municipality).

#### 3.6.2. Greater Recognition of Populations with Access and Functional Needs

Several CBOs emphasized the need to tailor services to the needs of people with particular disabilities. “Consider the population…with disabilities... They must have trained staff for people with different conditions such as autism, people with schizophrenia, and Alzheimer’s”. (Large non-profit organization serving people with disabilities).

This includes recognizing the importance of serving older adults and disabled people with intersectional identities:

Take into consideration all intersections of people, communities with undefined immigration status, LGBTQ, lack of access to services, take into consideration these populations that are excluded from emergency management. Accessibility of supplies, distribution of supplies, unnecessary bureaucracy, the process needs to be streamlined so that people do not die. (Medium-sized organization serving women).

##### Prioritizing the Elderly during the COVID-19 Pandemic

A small non-profit organization serving the elderly stated that the elderly were not receiving the vaccine prioritization they deserved during the COVID-19 pandemic, placing them at greater risk. The KI mentioned that “In the pandemic, the same thing happened again with the hurricane, where they did nothing for them [the elderly]. We returned to the same problem, in which we did not learn more. I believe that when things happen, we must learn”.

## 4. Discussion

CBOs responding to hurricanes, earthquakes, and the COVID-19 pandemic provided an impressively broad range of essential services, sometimes going beyond the original scope of the organization. This is particularly notable given that these CBOs continue to operate under severe financial, logistical, and resource constraints in a Puerto Rican context, with a lack of infrastructure. CBOs play a critical role in building social infrastructure, fostering community engagement, coalition-building with like-minded organizations, and calling for government transparency. These organizations, in serving older adults with disabilities, support their interdependence and agency, while also promoting a more inclusive society in which the capacity of all members is fostered.

People with disabilities often disproportionately experience other forms of vulnerability. This research deals with the intersection of age and disability, given that while older adults may be experiencing age-related functional impairment, there may also be older adults included among groups of younger people identified by a common disability such as deafness. Our findings illustrate that other identities impact their experiences, such as having mental health issues, having a low income, or having low literacy skills. Therefore, there is a multitude of ways in which the individuals served by these organizations are marginalized.

The findings show that organizations played an important role in serving often underserved subgroups of people with functional and access needs, including individuals facing homelessness or drug addiction, families in crisis or facing violence, and unemployed women. In response to Hurricane María and COVID-19, these organizations provided critical emergency response services simply by achieving their organization’s mission in providing referral services, food, employment assistance, and durable medical equipment.

The majority of organizations served people with low literacy 21/22 (95%), while 21/22 (95%) of the organizations served low-income people. This fills an important need given poverty levels in Puerto Rico and lower levels of education overall [28], problems which were further exacerbated by the COVID-19 pandemic. Since Hurricane María, hundreds of schools have closed across Puerto Rico, limiting children’s access to education, and austerity measures imposed by PROMESA have only worsened poverty [29].

During COVID-19, providing food distribution to the elderly reduced their risk of contracting the disease. In addition, offering job training allowed these subgroups to sustain themselves and maintain the health of their families. It is important to recognize that the emergency response role of these organizations goes beyond what traditional emergency management might categorize as emergency response. These organizations are positioned to enable emergency recovery in the long term, beyond the immediate crisis. Due to the ongoing nature of the COVID-19 pandemic, providing sufficient resources and bandwidth for these organizations is more critical than ever. It is imperative we highlight the importance of government accountability and support as these organizations step up to the plate when the government fails to respond efficiently.

In response to a question about how organizations intended to serve their clients in future emergencies, it is heartening to see the wide range of services they are committed to providing. However, few organizations reported intending to provide relatively low-cost interventions such as first aid and cardiopulmonary resuscitation (CPR) (9/22) or establishing a buddy system (13/22). It is important to incentivize organizations to provide these low-cost interventions as they can save lives. Further, only half of the organizations intended to provide interpretation for the deaf and hard of hearing (11/22). Despite resource constraints, it is important for organizations to think of ways to be accessible for deaf and hard-of-hearing Puerto Ricans. Even if providing interpretation may have associated costs, perhaps there are alternative methods of undertaking outreach with deaf communities. Nevertheless, CBOs can take into account the particular needs of certain people in the population they serve, and can refer them to other organizations as needed.

With the surge of the COVID-19 pandemic, CBOs have faced additional challenges, as the risk and impact of the pandemic remains high for at-risk populations including the elderly and individuals with access and functional needs [30]. COVID-19 has disrupted the daily lives of individuals who rely on CBOs for services and support. Hence, CBOs play a vital role in delivering services to these individuals while allowing them to adjust to the way of life during the pandemic.

This study provides information about CBOs geared to serving the elderly, individuals with disabilities, and individuals with mental illness. The findings provide evidence for the value of these organizations in the response and recovery efforts at the community level, as well as areas that need improvement in serving older adults and disabled populations. Most of the people harmed in disasters are older adults and people with disabilities. We have also seen the impact on these populations during the COVID-19 pandemic. Older adults and people of any age who have serious underlying medical conditions (e.g., heart or lung disease or diabetes) are at higher risk of developing serious complications from COVID-19 [31,32]. People with disabilities may experience more difficulty in engaging in preventative efforts such as social distancing [33], and they may experience problems related to wearing masks, losing access to caregivers or support networks due to social distancing, or inaccessible telehealth options or testing sites [34,35], as well as disruptions to the health services they normally rely on [36]. The COVID-19 experience provides an example of the limitations of preventive efforts or emergency management for people with disabilities and older adults. As discussed in the Introduction, during Hurricane María, older adults and people with disabilities were impacted by inadequate government response efforts. Having an emergency plan in order to continue operating through emergencies, or resuming operations as quickly as possible, is crucial for the survival and well-being of at-risk populations.

Our findings suggest gaps on multiple levels for CBOs: the financial support needed to serve these populations, training on serving populations with access and functional needs, and mental health support for staff. Research indicates the pervasive impact of COVID-19 on employees’ mental health [37]. Previous research on Hurricane Katrina illustrated the challenges service providers frequently face, such as lack of adequate training and preparedness in working with trauma-exposed displaced evacuees. As can be imagined, lack of training can be a significant barrier to providing care [24,25]. Furthermore, findings demonstrate that some CBOs are prioritizing training in recovery and response while excluding training in preparedness, which can minimize the damage done by emergencies.

Interview data from interviews with older adults [38], along with the data from the CBO surveys discussed in this paper, can contribute towards improving emergency response plans, including accessible evacuation and communications. These organizations serve as the first line of defense, along with federal efforts; however, unlike FEMA, these organizations are familiar with the local population’s needs and specific cultural nuances.

These data also identified the pivotal need for governmental agencies and CBOs to support older adults with disabilities given the current political and social infrastructure in Puerto Rico. Research demonstrates that FEMA’s response to the hurricane was limited compared to the response to similar types of disasters that occurred in the mainland United States [39,40]. The potential for inequity is hard to ignore given that Puerto Rico is an unincorporated territory of the United States and does not have benefits afforded to the states in the union.

### 4.1. Mental Health

Some participants in our study reported insufficient training as an important barrier to providing adequate mental health services [25]. People in a disaster, emergency, or traumatic event will experience high levels of psychological distress [41,42,43,44]. Within an emergency response, particular attention must be paid to the mental health needs of at-risk populations such as older adults and people with disabilities. Some studies discuss the high incidence of mental health issues in the aftermath of Hurricane María and COVID-19 [45,46].

CBOs have the potential to mitigate the stress produced by an emergency. In fact, social workers and mental health professionals working within these organizations played a major role in supporting the mental health care needs of their local communities after the hurricane [47,48]. Research found that psychological first aid, as well as a community-based psychosocial support structure, was integrated into the National Disaster Response Plan of Puerto Rico [49].

People working in CBOs frequently provide mental health care despite experiencing the “shared trauma” of a disaster [50]. A key finding in our study was the need for mental health support for staff at Puerto Rican CBOs, which supports research documenting burnout among Puerto Rican health care staff during COVID-19 [51]. These organizations are providing essential services and basic needs support to their local communities while also living through the same humanitarian crisis. Federal efforts must contribute to supporting these individuals working in these organizations so that they can undertake their work effectively. Previous research discusses the need to provide disaster response strategies and information in order to prevent mental health issues. [52]. Given our findings, we would add that it is necessary to include the professionals and service providers involved in recovery efforts. Previous research discusses the need for health care and social service providers, but these efforts must incorporate those working specifically in nonprofit organizations or non-governmental agencies which may take on as much or more disaster relief during a disaster than government agencies [53].

### 4.2. Coalition Building

More coordination and collaboration (partnering) between organizations during emergencies are necessary. Networks that connect these organizations should share recovery models, creating a more robust emergency response through strategic collective action, consulting with each other on areas of need, and sharing response efforts. Previous research provides examples of successful collaborations for post-disaster response and recovery [54]. A toolkit developed by the U.S. Department of Health and Human Services provides information on how to specifically support older adults and people with disabilities, which organizations could use to improve their focus on these populations [55]. Resources like these should be used, but a central network is needed that helps to distribute these resources and provides physical spaces, online platforms, and conferences to enable organizations to collaborate.

### 4.3. Recommendations for Improving the Emergency Operations Plan

CBOs had a wide variety of recommendations on including older adults and people with disabilities more effectively in the EOP. Most commonly cited was the desire for the state to facilitate and funnel resources and connect with CBOs. Enabling collective action and having a plan that includes CBO networks working in tandem with the government were also important. Research indicates that older adults with disabilities could be evacuated more effectively if there was a centralized surveillance system, which would only work if the government enlisted the help of CBOs to broaden its reach and impact [56].

Puerto Rico continues to have issues with infrastructure. The electrical grid failed during the hurricane, in part because of a lack of maintenance. The electricity grid has recently been privatized, yet there have been continual outages since the company took over, even in the absence of storms. Given this situation, the government could lead efforts to connect organizations better and could provide an overarching plan of action for disasters.

### 4.4. Improving Funding

In the years since Hurricane María, the cumulative effects of Puerto Rico’s unique status as a territory and multiple and ongoing disasters remain evident. More funding opportunities at the federal level, specifically for training, salaries, resources, and provision of services are necessary. Government funding schemes should be made more accessible to CBOs that have direct ties to the community they serve and are most in need of support [57].

### 4.5. Future Research

Given the relatively small response rate of participating CBOs, our study warrants further research. It is necessary to develop a larger, quantitative study based in Puerto Rico with academics and health care professionals living there that provides key findings on characteristics of organizations and all levels of work related to emergency preparation, management, and response. It is possible that other types of data collection methods such as focus groups with organization leaders could help develop a coalition that better collaborates in responding to disasters. Incorporating embodied knowledge on the ground while decolonizing methods could foster empowerment [58].

Future research could consider how these multiple intersecting identities influence experiences after a disaster and how CBOs can best address the needs of people with intersecting identities, despite a CBO’s mission focusing on one particular identity.

## 5. Conclusions

Disability studies scholars have conceptualized ecosystem impairments, both natural and human-made, as parts of larger networks of disabled ecologies connected to limitations, interdependence, and adaptation [59]. This reflects how Puerto Rico, as the site of ongoing disasters, has enabled people with disabilities, by necessity, to become interdependent with their community networks, including CBOs. CBOs have, to their credit, adapted to that climate. In addition to the ongoing effects of the COVID-19 crisis, Hurricane María, and recurring hurricanes and earthquakes, CBOs in Puerto Rico are operating within a state of continual emergency [2].

Moreover, while also providing emergency preparedness and response services to their staff and communities, the majority of CBOs are serving subgroups of older adults and people with disabilities who are individually experiencing multiple and overlapping crises, including mental health challenges, food insecurity, homelessness, domestic violence, family crises, and lack of employment or low-income status. Therefore, these populations served by Puerto Rican CBOs are particularly at risk of living in a state of continual crisis due to pandemics or natural disasters [8].

Puerto Rican CBOs play a critical role in EPR or disability-inclusive disaster risk reduction (DiDRR) for communities living with heightened medical and functional needs such as individuals with disabilities or mental illness and the elderly [9]. In Puerto Rico, it is imperative that emergency management officials, CBOs, and communities in crisis receive sufficient resources and mental health support. To achieve this, better integration of the needs of older adults and people with disabilities into the Emergency Operations Plan (EOP) is also required.

## Figures and Tables

**Table 1:** Ways organizations assisted clients with the evacuation process after Hurricane María.

Strategies	Frequency (*n* = 22)	Percentage
Information or referral services	16	72.7%
Outreach to clients in the home or neighborhood	15	68.1%
Food needs	13	59%
Emergency or disaster updates	13	59%
Communication links between family members	13	59%
Durable medical equipment	10	45.4%
Disaster kit (emergency/disaster kit)	9	40.9%
Transportation	8	36.3%
Interpretation or translation	6	27.2%
Medical or health care	5	22.7%
Administering medication	1	4.5%
Other	3	13.6% ^1^
Organization does not engage in these activities	1	N/A ^2^

^1^ Mental health, diabetes monitoring, distributing basic medical necessities to the elderly. ^2^ Organization established after Hurricane María.

**Table 2:** Ways organizations plan to check on and care for clients after a future emergency.

Strategies	Frequency (*n* = 22)	Percentage
Providing food	21	95.5%
Making phone/video calls	19	86.3%
Using social media (i.e., Twitter, Facebook)	18	81.8%
Providing information/referrals for services	18	81.8%
Conducting outreach in the home or neighborhood	17	77.2%
Sending text messages	16	72.7%
Sending emails	15	68.1%
Establishing a buddy system	13	59%
Providing transportation	12	54.5%
Providing interpretation for the deaf and hard of hearing	11	50%
Providing shelter or alternative housing	10	45.4%
Providing first aid or CPR	9	40.9%

**Table 3:** Actions needed to improve services to disabled ^1^ and older clients in a future emergency.

Actions	Frequency (*n* = 20)	Percentage
Increase availability of supplies and equipment	17	85%
Improve emergency preparedness training for organization staff	16	80%
Improve emergency preparedness training for clients served by the organization	16	80%
Improve emergency preparedness education materials—availability of alternative formats (large print, video (with ASL/captioning), audio, Braille)	16	80%
Increase number of staff devoted to emergency preparedness	15	75%
Increase financial resources	13	65%
Improve emergency preparedness education materials—availability of other languages	13	65%
Improve emergency preparedness education materials—simplify by using “plain language”	3	15%

^1^ This question specified the following groups: the elderly, deaf, blind, physically disabled, and developmentally disabled, and people with mental health conditions. One organization had no recommendations and one did not answer the question.
